# Notch-mediated hepatocyte MCP-1 secretion causes liver fibrosis

**DOI:** 10.1172/jci.insight.165369

**Published:** 2023-02-08

**Authors:** Jinku Kang, Jorge Postigo-Fernandez, KyeongJin Kim, Changyu Zhu, Junjie Yu, Marica Meroni, Brent Mayfield, Alberto Bartolomé, Dianne H. Dapito, Anthony W. Ferrante, Paola Dongiovanni, Luca Valenti, Remi J. Creusot, Utpal B. Pajvani

**Affiliations:** 1 Department of Medicine, Naomi Berrie Diabetes Center, and; 2 Columbia Center for Translational Immunology, Columbia University Irving Medical Center, New York, New York, USA.; 3 Department of Biomedical Sciences, College of Medicine, Program in Biomedical Science & Engineering, and Research Center for Controlling Intercellular Communication (RCIC), Inha University, Incheon, South Korea.; 4 Memorial Sloan Kettering Cancer Center, New York, New York, USA.; 5 General Medicine and Metabolic Diseases, Fondazione IRCCS Cà Granda Ospedale Maggiore Policlinico, Milan, Italy.; 6 Instituto de Investigaciones Biomédicas Alberto Sols (CSIC/UAM), Madrid, Spain.; 7 Department of Pathophysiology and Transplantation, Università degli Studi di Milano, Milan, Italy.; 8 Precision Medicine Lab, Biological Resource Center, Department of Transfusion Medicine and Hematology, Fondazione IRCCS Cà Granda Ospedale Maggiore Policlinico Milan, Milan, Italy.

**Keywords:** Gastroenterology, Metabolism, Chemokines, Fibrosis

## Abstract

Patients with nonalcoholic steatohepatitis (NASH) have increased expression of liver monocyte chemoattractant protein-1 (MCP-1), but its cellular source and contribution to various aspects of NASH pathophysiology remain debated. We demonstrated increased liver *CCL2* (which encodes MCP-1) expression in patients with NASH, and commensurately, a 100-fold increase in hepatocyte *Ccl2* expression in a mouse model of NASH, accompanied by increased liver monocyte-derived macrophage (MoMF) infiltrate and liver fibrosis. To test repercussions of increased hepatocyte-derived MCP-1, we generated hepatocyte-specific *Ccl2*-knockout mice, which showed reduced liver MoMF infiltrate as well as decreased liver fibrosis. Forced hepatocyte MCP-1 expression provoked the opposite phenotype in chow-fed wild-type mice. Consistent with increased hepatocyte Notch signaling in NASH, we observed a close correlation between markers of Notch activation and *CCL2* expression in patients with NASH. We found that an evolutionarily conserved Notch/recombination signal binding protein for immunoglobulin kappa J region binding site in the *Ccl2* promoter mediated transactivation of the *Ccl2* promoter in NASH diet–fed mice. Increased liver MoMF infiltrate and liver fibrosis seen in opposite gain-of-function mice was ameliorated with concomitant hepatocyte *Ccl2* knockout or CCR2 inhibitor treatment. Hepatocyte Notch activation prompts MCP-1–dependent increase in liver MoMF infiltration and fibrosis.

## Graphical abstract

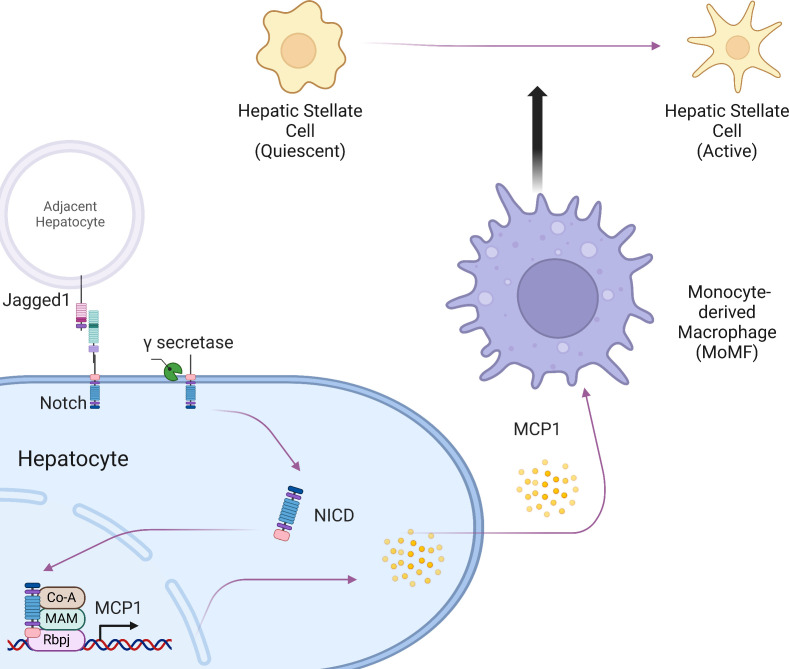

## Introduction

Nonalcoholic fatty liver disease (NAFLD) is one of the most common metabolic complications of obesity (1). NAFLD is defined by excess hepatic lipid content (2) but often remains clinically silent in the absence of hepatocyte injury and inflammation that are associated with nonalcoholic steatohepatitis (NASH), which in turn may lead to fibrosis (3). NASH has no approved pharmacotherapy, in part due to incomplete understanding of molecular drivers of NASH-induced liver fibrosis, the major determinant of mortality in these patients (4).

NASH is defined by the presence of inflammatory cells in the liver lobule (5), including increased numbers of both liver-resident macrophages (Kupffer cells) and monocyte-derived macrophages (MoMFs) (6). One possible contributor to MoMF infiltrate is increased expression of the chemokine monocyte chemoattractant protein-1 (MCP-1). MCP-1 recruits and activates monocytes to the site of tissue injury and regulates adhesion molecules and pro-inflammatory cytokines (7). Serum MCP-1 is associated with NASH severity (8) and risk of progression to cirrhosis (9). Consistent with MCP-1’s role in MoMF recruitment (10), inhibition of MCP-1 cognate chemokine receptors such as CCR2 reduces liver macrophage content (11), with preferential effects on liver MoMF infiltrate. These data suggest that MCP-1 is a major determinant of MoMF recruitment in NASH (12, 13).

Multiple liver cell types express and release MCP-1 in response to a variety of upstream signals. For instance, increased activation of Toll-like receptors in Kupffer cells and hepatic stellate cells (HSCs) can lead to increased MCP-1 secretion (14, 15). Secretion of MCP-1 and other chemokines from these nonparenchymal cells (NPCs) increases recruitment of inflammatory cells to damaged liver (16) and regulates the wound-healing response (17, 18), but we hypothesized that hepatocyte secretion of chemokines may represent an early response to parenchymal injury. Indeed, MCP-1 secretion from hepatocytes has also been demonstrated (19), but the relative contribution of hepatocyte-derived and NPC-derived MCP-1 to NASH pathogenesis, and the mechanisms underlying MCP-1 expression in hepatocytes, remain largely unknown. Here, we report our findings that hepatocyte MCP-1 was markedly increased in mice fed a NASH-provoking diet, due to pathologic increase in hepatocyte Notch activity (20), which led to increased MoMF infiltrate and liver fibrosis.

## Results

### Hepatocyte MCP-1 is increased in mice fed a NASH-provoking diet.

**Figure 1:**
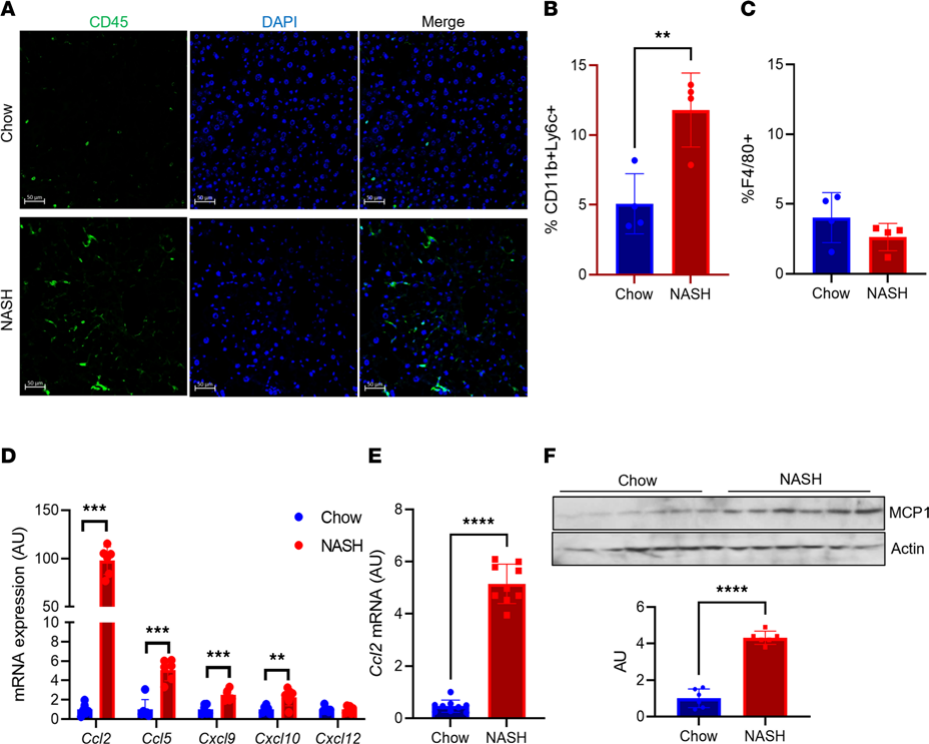
Liver MCP-1 expression is increased in NASH diet–fed mice. (**A**) Representative images of CD45^+^ cells in livers from chow- and NASH diet–fed wild-type (WT) male C57BL/6J mice. (**B**) FACS analysis of CD11b^+^Ly6C^+^ and (**C**) CD11b^+^F4/80^+^ cells from nonparenchymal cells (NPCs) isolated from chow- and NASH diet–fed WT male mice (*n* = 4 mice/group). (**D**) Gene expression of key chemokines in hepatocytes isolated from chow- and NASH diet–fed WT male mice (*n* = 6 mice/group). (**E**) *Ccl2* gene expression in whole liver from chow- and NASH diet–fed WT male mice (*n* = 9 mice/group). (**F**) MCP-1 protein and quantitation in whole liver from chow- and NASH diet–fed WT male mice (*n* = 6 mice/group). MCP-1 and actin blots are derived from the same samples run contemporaneously in parallel gels. Scale bars: 50 μm. All data are shown with group means ± SEM; **, *P* < 0.01, ***, *P* < 0.001, ****, *P* < 0.0001 by 2-tailed *t* test.

Liver immune cell number was increased in wild-type (WT) mice fed a NASH-provoking diet rich in palmitate, sucrose, and cholesterol, coupled to ad libitum access to fructose-containing drinking water (Figure 1A), which leads to increased liver lipid content (Supplemental Figure 1, A and B; supplemental material available online with this article; https://doi.org/10.1172/jci.insight.165369DS1) and fibrosis (21). To define the immune cell infiltrate in this model, we isolated liver NPCs from WT mice fed normal chow or NASH diet for 16 weeks, then applied flow cytometry analysis. After gating with anti-CD45, we found large relative changes in several immune cell populations (Supplemental Table 1). We focused on CD11b^+^ macrophage populations, which we defined as resident Kupffer cells or MoMFs, based on low or high Ly6C (6) expression, respectively (Supplemental Figure 1C). While Kupffer cell percentage among total CD45^+^ cells was unchanged, we observed increased numbers and percentage of MoMFs in NASH diet–fed mice (Figure 1, B and C; Supplemental Table 1; and Supplemental Figure 1, D and E).

To evaluate potential mechanisms of increased MoMF infiltrate in NASH, we isolated hepatocytes from chow- and NASH diet–fed WT mice. We observed increased expression of multiple chemokines in response to NASH diet feeding, most prominently *Ccl2* (Figure 1D and Supplemental Figure 1, F and G), which encodes MCP-1. We found commensurately increased *Ccl2* in livers from NASH diet–fed WT mice by quantitative PCR (qPCR) and Western blot (Figure 1, E and F). These data suggest that hepatocyte-derived MCP-1 may affect the liver microenvironment in NASH.

### Hepatocyte-derived MCP-1 promotes liver fibrosis.

**Figure 2:**
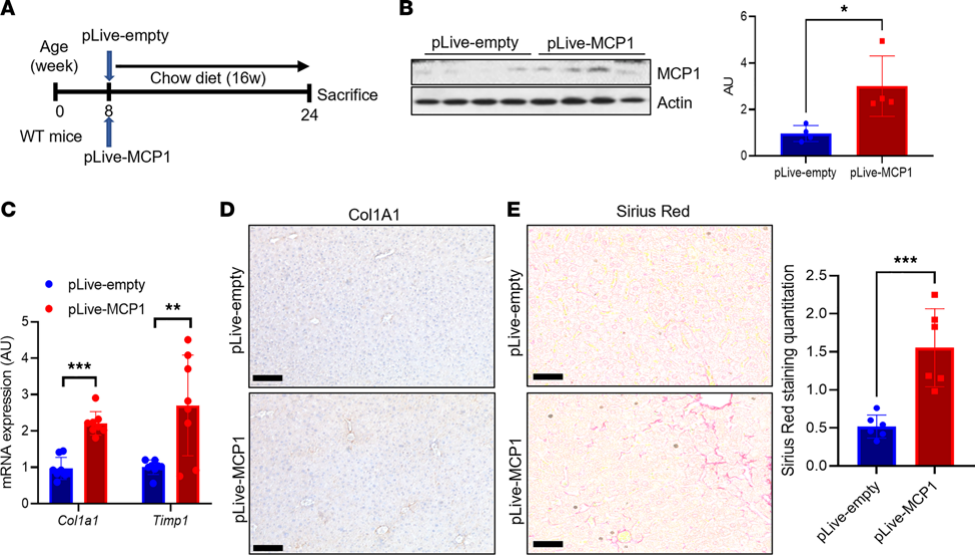
Hepatocyte-derived MCP-1 is necessary and sufficient to induce liver fibrosis. (**A**) Experimental schematic for hepatocyte-specific MCP-1 gain of function by hydrodynamic injection of control (pLive-empty) or MCP-1 (pLive-MCP1) vectors in WT male mice (*n* = 8 mice/group). (**B**) Liver MCP-1 protein and quantitation from hepatocyte-specific MCP-1 gain of function by hydrodynamic injection of control (pLive-empty) or MCP-1 (pLive-MCP1) vectors in WT male mice (*n* = 4 mice/group). MCP-1 and actin blots are derived from the same samples run contemporaneously in parallel gels. (**C**) Gene expression for markers of hepatic stellate cell (HSC) activity from hepatocyte-specific MCP-1 gain of function by hydrodynamic injection of control (pLive-empty) or MCP-1 (pLive-MCP1) vectors in WT male mice (*n* = 8 mice/group). (**D**) Representative IHC image of Col1a1 protein expression from hepatocyte-specific MCP-1 gain of function by hydrodynamic injection of control (pLive-empty) or MCP-1 (pLive-MCP1) vectors in WT male mice. (**E**) Sirius red staining and quantitation from hepatocyte-specific MCP-1 gain of function by hydrodynamic injection of control (pLive-empty) or MCP-1 (pLive-MCP1) vectors in WT male mice (*n* = 6 mice/group). Scale bars: 50 μm. All data are shown with group means ± SEM; *, *P* < 0.05, **, *P* < 0.01, ***, *P* < 0.001 by 2-tailed *t* test.

We next tested repercussions of increased hepatocyte-derived MCP-1 in NASH, using both gain- and loss-of-function approaches. First, we performed hydrodynamic injection of an albumin promoter-driven MCP-1–encoding plasmid (Figure 2A), which resulted in a similar increase in liver MCP-1 as seen in NASH diet–fed mice (Figure 2B), which we attributed to but did not confirm as hepatocyte-specific expression. Forced MCP-1 expression led to no difference in body weight, liver or epididymal white adipose tissue (eWAT) weight, blood glucose, liver triglyceride (TG), or cholesterol (Supplemental Figure 2, A–F) and aside from modest increases in *Itgam* (which encodes CD11b) did not significantly change gene expression corresponding to other immune cells (Supplemental Figure 2G). Nevertheless, we observed increased markers of HSC activity that translated to higher liver staining of collagen type I alpha 1 chain (Col1A1), the predominant liver collagen. This translated to an approximately 3-fold increase in liver fibrosis by Sirius red staining (Figure 2, C–E), suggesting that increase in hepatocyte-derived MCP-1 is sufficient to promote liver fibrosis even in lean mice.

**Figure 3:**
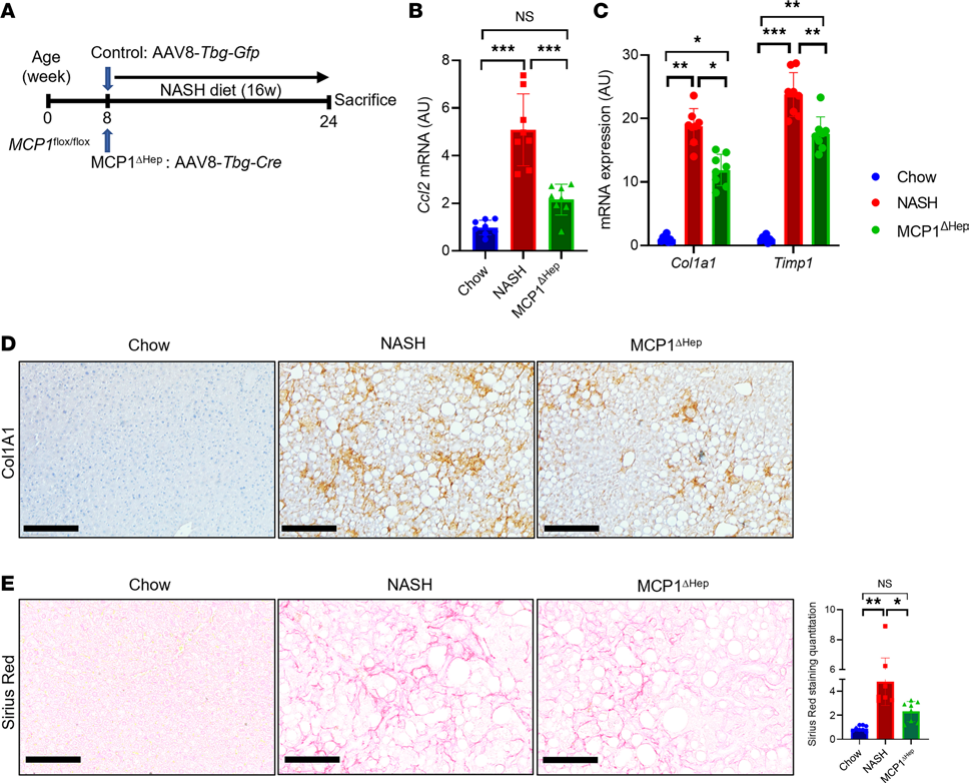
MCP-1 loss of function reduces liver fibrosis with NASH diet for 16 weeks. (**A**) Experimental schematic for hepatocyte-specific MCP-1-knockout mice. Male 8-week-old MCP-1^fl/fl^ mice were transduced with AAV8-*Tbg-Gfp* (Control) or AAV8-*Tbg-Cre* to generate MCP-1^ΔHep^ male mice, then fed with NASH diet for 16 weeks (*n* = 8 mice/group). (**B**) Gene expression for liver *Ccl2* from chow- or NASH diet–fed control mice or MCP-1^ΔHep^ male mice (*n* = 8 mice/group). (**C**) Markers of HSC activity from chow- or NASH diet–fed control mice or MCP-1^ΔHep^ male mice (*n* = 8 mice/group). (**D**) Representative IHC image of Col1a1 protein expression from chow- or NASH diet–fed control mice or MCP-1^ΔHep^ male mice. (**E**) Liver Sirius red staining and quantitation in control and MCP-1^ΔHep^ male mice (*n* = 8 mice/group). Scale bars: 50 μm. All data are shown with group means ± SEM; *, *P* < 0.05, **, *P* < 0.01, ***, *P* < 0.001 by 1-way ANOVA followed by Tukey’s multiple comparisons test.

Next, to test the necessity of hepatocyte MCP-1 for NASH diet–induced inflammation and fibrosis, we generated hepatocyte-specific MCP-1–knockout (MCP-1^ΔHep^) mice by transducing MCP-1–floxed animals with AAV8-*Tbg*-*Cre*, which takes advantage of liver targeting of AAV8 and the hepatocyte-specific *thyroxine binding globulin* (*Tbg*) promoter (22) (Figure 3A). NASH diet-fed MCP-1^ΔHep^ mice showed no difference in body or liver weight, adiposity, blood glucose, liver TG, or cholesterol (Supplemental Figure 3, A–F). However, ablation of hepatocyte-derived MCP-1 largely suppressed NASH diet–induced increase in liver *Ccl2* (Figure 3B) and partially abrogated HSC activation and liver fibrosis (Figure 3, C–E). MCP-1^ΔHep^ mice showed reduced liver *Itgam* expression, but again, no substantive changes in expression of markers of other liver immune cells (Supplemental Figure 3G). In sum, these data suggest that increase in hepatocyte-derived MCP-1 is both necessary and sufficient for HSC activity and NASH-induced liver fibrosis.

### Hepatocyte MCP-1 is determined by Notch activity.

**Figure 4:**
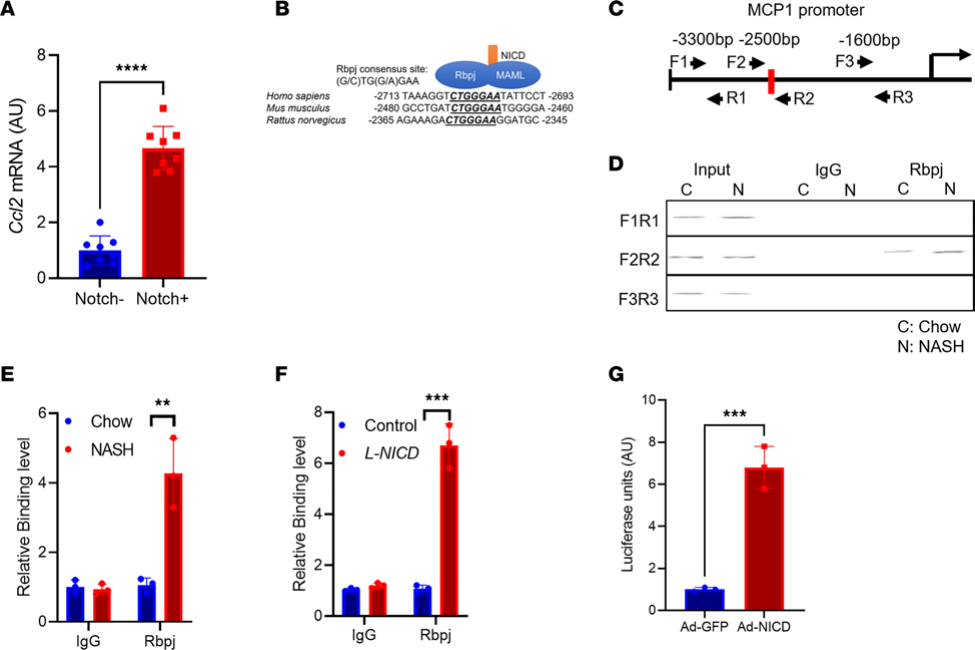
Hepatocyte Notch activity regulates MCP-1. (**A**) *Ccl2* expression in Notch-inactive and -active hepatocytes isolated from NASH diet–fed transgenic Notch reporter male mice (*n* = 8 mice/group). (**B**) Comparative sequence alignment of MCP-1 promoter, with evolutionarily conserved Rbpj binding site indicated. (**C**) Experimental schematic for chromatin immunoprecipitation (ChIP) experiment, showing primer pairs that include (F2/R2) or bind outside (F1/R1 and F3/R3) the Rbpj binding site in the MCP-1 promoter. (**D** and **E**) Rbpj occupancy at the MCP-1 promoter in livers from chow- and NASH diet–fed WT mice (*n* =3 mice/group). (**F**) Rbpj occupancy at the MCP-1 promoter in livers from Cre- control and hepatocyte-specific Notch gain-of-function (*L-NICD*) male mice (*n* = 3 mice/group). (**G**) MCP-1 promoter-luciferase activity from Ad-GFP and Ad-NICD transduction of mouse primary hepatocytes (*n* = 3 biologic replicates/group). All data are shown with group means ± SEM; **, *P* < 0.01, ***, *P* < 0.001, ****, *P* < 0.0001 by 2-tailed *t* test.

**Figure 5:**
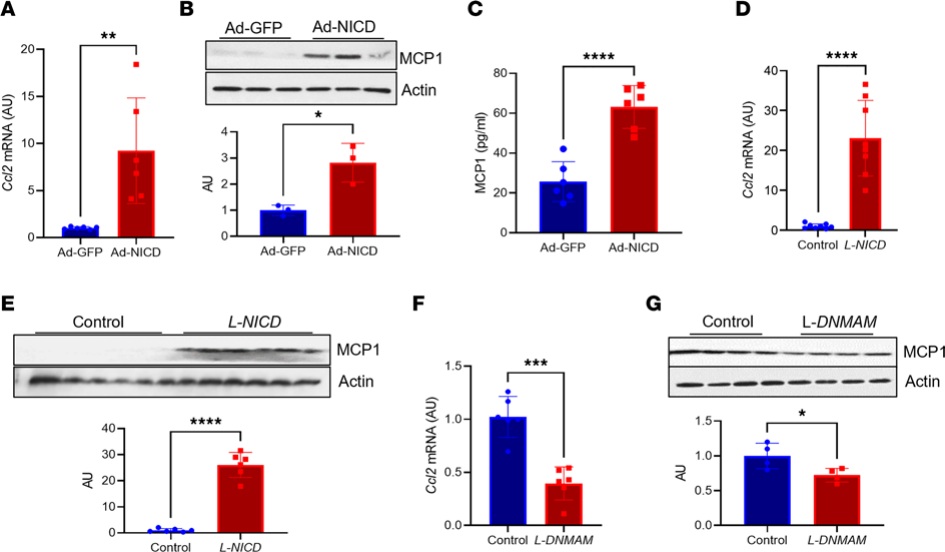
Notch gain or loss of function reduces hepatocyte MCP-1 levels. (**A**) *Ccl2* gene expression in WT primary hepatocytes transduced with adenovirus encoding GFP or NICD (*n* = 6 biologic replicates/group). (**B**) MCP-1 protein in WT primary hepatocytes transduced with adenovirus encoding GFP or NICD (*n* = 3 biologic replicates/group). MCP-1 and actin blots are derived from samples run on the same gel, with filter paper cut and probed separately. (**C**) Circulating MCP-1 in WT primary hepatocytes transduced with adenovirus encoding GFP or NICD (*n* = 6 biologic replicates/group). (**D**) Liver *Ccl2* gene expression in Cre- and *L-NICD* male mice (*n* = 8 mice/group). (**E**) MCP-1 protein levels in Cre- and *L-NICD* male mice (*n* = 6 mice/group). MCP-1 and actin blots are derived from samples run on the same gel, with filter paper cut and probed separately. (**F**) *Ccl2* gene expression in livers from NASH diet–fed Cre- and hepatocyte-specific Notch loss-of-function (*L-DNMAM*) male mice (*n* = 6 mice/group). (**G**) MCP-1 protein levels in livers from NASH diet-fed Cre- and hepatocyte-specific Notch loss-of-function (*L-DNMAM*) male mice (*n* = 4 mice/group). MCP-1 and actin blots are derived from the same samples run contemporaneously in parallel gels. All data are shown with group means ± SEM; *, *P* < 0.05, **, *P* < 0.01, ***, *P* < 0.001, ****, *P* < 0.0001 by 2-tailed *t* test.

We next turned our attention to the mechanism of increased hepatocyte MCP-1 in NASH. Intriguingly, RNA sequencing of hepatocytes (23) isolated from NASH diet–fed transgenic Notch reporter mice (24) revealed a 5-fold increase in *Ccl2* expression in Notch-active (indicated by Venus reporter expression) versus -inactive hepatocytes (Figure 4A). As hepatocyte Notch signaling is activated in patients with NASH or in NASH diet feeding in mice (20, 25), we investigated whether Notch directly or indirectly regulates hepatocyte *Ccl2* expression. Notch activity requires generation of a Notch intracellular domain/mastermind-like/recombination signal binding protein for immunoglobulin kappa J region (NICD/MAML/Rbpj) transcriptional complex, which binds and transactivates promoter elements (26). We identified an evolutionarily conserved Rbpj consensus binding site (27) in the *Ccl2* promoter (Figure 4B). To test if *Ccl2* is a direct downstream transcriptional target of Notch, we determined Rbpj occupancy on the hepatocyte *Ccl2* promoter using a ChIP-Seq–validated anti-Rbpj antibody (28). We designed primer pairs that did or did not contain the putative Rbpj binding site (Figure 4C) and performed ChIP from livers of mice fed normal chow or NASH diet for 16 weeks, which revealed that Rbpj occupancy of the *Ccl2* promoter was significantly increased in the presence of endogenous Notch activity (Figure 4, D and E). Similarly, we observed increased Rbpj binding to this same promoter element in chow diet–fed hepatocyte-specific Notch gain-of-function (*L-NICD*) mice, generated by transducing NICD-floxed animals with AAV8-*Tbg*-*Cre* (Figure 4F). To test the functional consequence of Rbpj binding, we generated a luciferase construct containing the proximal 3 kb of the mouse *Ccl2* promoter. We transfected this construct in mouse primary hepatocytes, with or without adenoviral NICD (Ad-NICD) transduction, and found that Notch activity increased *Ccl2* promoter–driven luciferase (Figure 4G). Consistent with this observation, Ad-NICD transduction of mouse primary hepatocytes increased hepatocyte *Ccl2* expression, Notch activity, and MCP-1 protein by Western blot (Figure 5, A and B, and Supplemental Figure 4A), which led to increased MCP-1 secretion (Figure 5C).

We next queried whether hepatocyte Notch activity was necessary and/or sufficient for *Ccl2* expression in vivo. Consistent with in vitro results, chow-fed *L-NICD* mice had increased Notch activity, liver *Ccl2* expression, and MCP-1 levels as compared with control animals transduced with AAV8-*Tbg*-*Gfp* (Figure 5, D and E, and Supplemental Figure 4B). These data show that forced hepatocyte Notch activity can recapitulate effects of NASH diet feeding to raise hepatocyte MCP-1 levels. Next, we performed the converse experiment using hepatocyte-specific Notch loss-of-function (*L-DNMAM*) mice that leverage a transgenic dominant-negative MAML allele (29). NASH diet–fed *L-DNMAM* mice showed reduced hepatocyte Notch activity (Supplemental Figure 4C), as well as a marked reduction in liver *Ccl2* expression and liver MCP-1 levels (Figure 5, F and G). In sum, these data show that hepatocyte Notch activity determines increased hepatocyte-derived MCP-1 in NASH and that forced hepatocyte Notch activity can recapitulate effects of NASH diet feeding to raise hepatocyte MCP-1 levels.

### Liver CCL2 expression tracks with Notch activity in patients.

**Figure 6:**
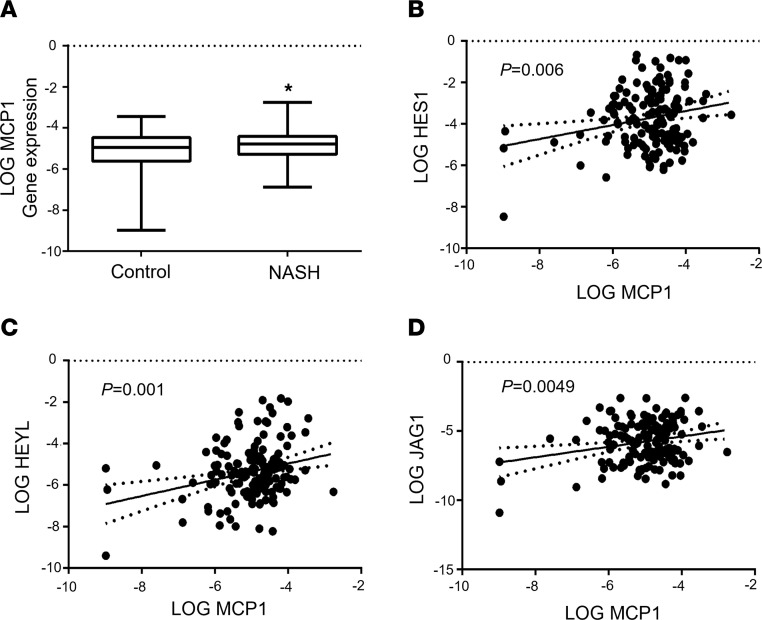
Liver *CCL2* expression tracks with NOTCH activity in patients. (**A**) Liver *CCL2* expression and correlation with canonical NOTCH targets (**B**) *HES1* and (**C**) *HEYL*, or upstream regulator (**D**) *JAG1*, as assessed by qPCR from liver biopsy in patients with (*n* = 63) versus without NASH (*n* = 82). Expression of all genes was log-transformed to ensure the assumption of normal distribution. All data are shown with group means ± SEM; *, *P* < 0.05 by 1-way ANOVA followed by Tukey’s multiple comparisons test.

To test whether these observations in mice translate to humans, we analyzed liver *CCL2* expression in liver biopsies of patients with suspected NASH. As observed in other cohorts (8, 30, 31), we found higher liver *CCL2* in patients with NASH than without NASH (Figure 6A), albeit with significant heterogeneity. We hypothesized that patients with higher liver Notch activity would show increased *CCL2*. As such, we assessed liver Notch activity by expression of canonical transcriptional targets *Hairy and Enhancer of Split 1* (*HES1*) and *Hairy/enhancer-of-split related with YRPW motif-like protein* (*HEYL*) and found a positive relationship between both and liver *CCL2* expression (Figure 6, B and C). *CCL2* expression was similarly associated with *JAG1*, which encodes JAGGED1, the ligand necessary for Notch receptor activity in liver (25) (Figure 6D). These data suggest that the relationship between hepatocyte Notch and MCP-1 identified in mice may extrapolate to patients with NASH.

### Hepatocyte-specific deletion of MCP-1 protects from Notch-induced fibrosis.

**Figure 7:**
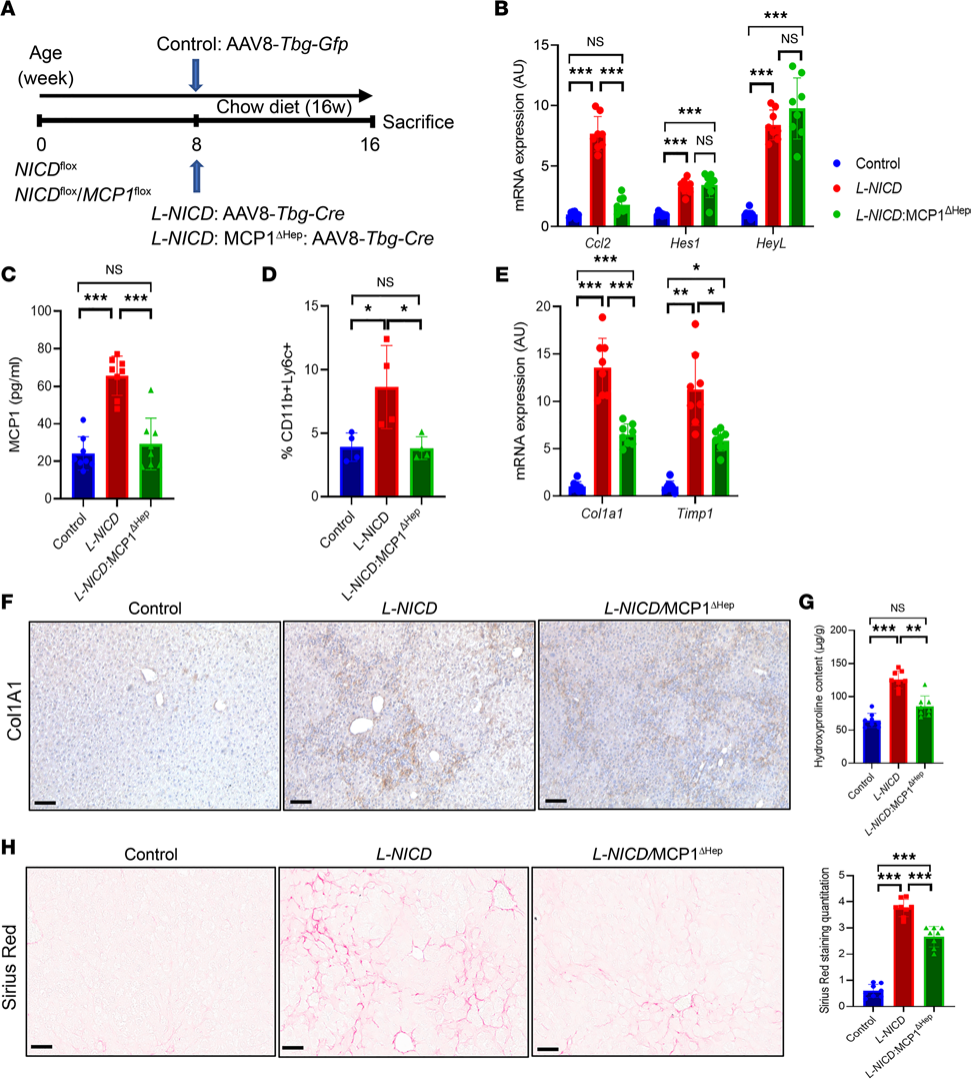
Notch-induced MCP-1 drives profibrotic macrophage infiltration and liver fibrosis. (**A**) Chow-fed NICD^fl/fl^ and NICD^fl/fl^ MCP-1^fl/fl^ were transduced with AAV8-*Tbg-Gfp* or AAV8-*Tbg-Cre* to generate control, *L-NICD*, and *L-NICD* MCP-1^ΔHep^ male mice (*n* = 8 mice/group). (**B**) Liver *Ccl2* and Notch target gene expression and (**C**) serum MCP-1 levels in control, *L-NICD*, and *L-NICD* MCP-1^ΔHep^ male mice (*n* = 8 mice/group). (**D**) FACS analysis of nonparenchymal cells (NPCs) isolated from livers of control, *L-NICD*, and *L-NICD* MCP-1^ΔHep^ male mice (*n* = 4 mice/group). (**E**) Gene expression for markers of HSC activity from livers of control, *L-NICD*, and *L-NICD* MCP-1^ΔHep^ male mice (*n* = 8 mice/group). (**F**) Representative IHC image of Col1a1 protein expression from livers of control, *L-NICD*, and *L-NICD* MCP-1^ΔHep^ male mice. (**G**) Hydroxyproline content from livers of control, *L-NICD*, and *L-NICD* MCP-1^ΔHep^ male mice (*n* = 8 mice/group). (**H**) Liver Sirius red staining and quantitation in control, *L-NICD*, and *L-NICD* MCP-1^ΔHep^ male mice (*n* = 8 mice/group). Scale bar: 50 μm. All data are shown with group means ± SEM; *, *P* < 0.05, **, *P* < 0.01, ***, *P* < 0.001 by 1-way ANOVA followed by Tukey’s multiple comparisons test.

We have previously shown that hepatocyte Notch activity is necessary and sufficient for HSC activity and liver fibrosis (20), phenocopying hepatocyte-specific MCP-1 gain- and loss-of-function mice. Based on these similarities, we hypothesized that Notch effects may partially depend on increased hepatocyte MCP-1. This hypothesis was hinted at by increased MoMF infiltrate in *L-NICD* livers (Supplemental Figure 4, D and E), which paralleled increased *Ccl2* expression in these mice (Supplemental Figure 4F). But to test this more directly, we intercrossed MCP-1/NICD-floxed mice, then transduced with AAV8-*Tbg*-*Cre* to generate *L-NICD* MCP-1^ΔHep^. We used NICD/MCP-1-floxed mice transduced with AAV8-*Tbg*-*Gfp* as negative controls and *L-NICD* littermates as positive controls (Figure 7A). We observed no differences in body weight, liver or eWAT weight, blood glucose, liver TG, or cholesterol in any of the groups (Supplemental Figure 5, A–F). But increased liver *Ccl2* expression in *L-NICD* mice was negated with concomitant hepatocyte MCP-1 deletion, which translated to a reduction in circulating MCP-1 back to basal levels (Figure 7, B and C). With reduction of hepatocyte-derived MCP-1, we observed a near normalization of liver CD45^+^ cells seen in *L-NICD* mice (Supplemental Figure 5G), accounted for primarily by a reduction in Notch-induced MoMF liver infiltrate (Figure 7D) as other liver immune cell populations were minimally changed (Table 1). Finally, as in NASH diet–fed MCP-1^ΔHep^ mice, hepatocyte MCP-1 ablation significantly ameliorated Notch-induced HSC activation (Figure 7, E and F), leading to lower hydroxyproline content (Figure 7G) and liver Sirius red staining (Figure 7H). These results suggest that increased hepatocyte MCP-1 secretion is an important determinant of Notch-induced liver infiltration of MoMF and liver fibrosis.

### CCR2 antagonism ameliorates Notch-induced liver fibrosis.

**Figure 8:**
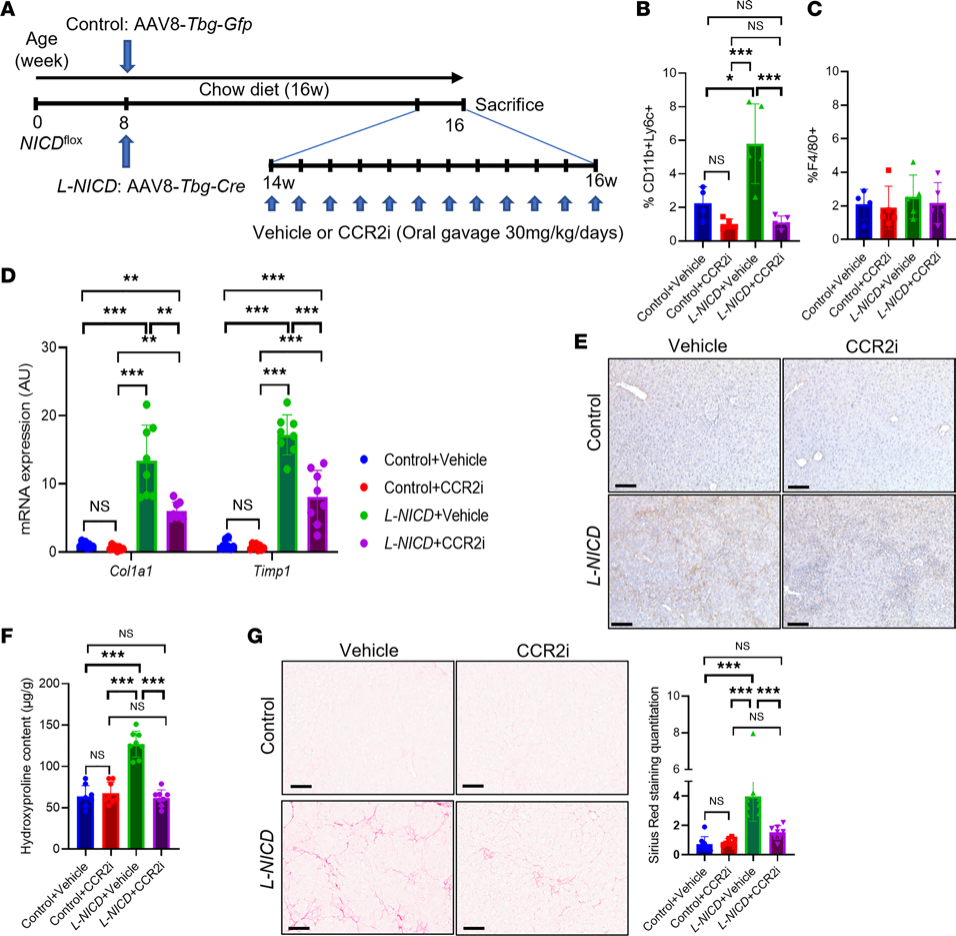
CCR2i treatment protects from Notch-induced liver fibrosis. (**A**) Chow-fed NICD^fl/fl^ male mice were transduced with AAV8-*Tbg-Gfp* or AAV8-*Tbg-Cre* to generate control and *L-NICD* mice, then treated with vehicle or CCR2i (30 mg/kg/d) by daily oral gavage for 2 weeks (*n* = 8 mice/group). (**B**) FACS analysis of CD11b^+^Ly6C^+^ and (**C**) F4/80^+^ nonparenchymal cells (NPCs) isolated from livers of control and *L-NICD* male mice treated with CCR2i or vehicle (*n* = 4–5 mice/group). (**D**) Gene expression for markers of HSC activity in control and *L-NICD* male mice treated with CCR2i or vehicle (*n* = 8 mice/group). (**E**) Representative IHC image of Col1a1 protein expression in control and *L-NICD* male mice treated with CCR2i or vehicle. (**F**) Hydroxyproline content in control and *L-NICD* male mice treated with CCR2i or vehicle (*n* = 8 mice/group). (**G**) Liver Sirius red staining and quantitation in control and *L-NICD* male mice treated with CCR2i or vehicle (*n* = 8 mice/group). Scale bar: 50 μm. All data are shown with group means ± SEM; *, *P* < 0.05; **, *P* < 0.01; ***, *P* < 0.001 by 1-way ANOVA followed by Tukey’s multiple comparisons test.

To assess the therapeutic implication of these findings, we administered a CCR2 inhibitor (CCR2i), which antagonizes the cognate receptor for the MCP-1 chemokine, to mice with advanced Notch-induced fibrosis by daily oral gavage for 2 weeks prior to sacrifice (Figure 8A). CCR2i treatment did not change body weight, liver or eWAT weight, blood glucose, or liver lipid content as compared to vehicle-treated mice (Supplemental Figure 6, A–F). But like hepatocyte MCP-1 deletion, CCR2i treatment reduced Notch-induced MoMF infiltrate back to control levels, without substantively affecting Kupffer cell number (Figure 8, B and C) or total liver immune cell number or subpopulations (Supplemental Figure 6G and Table 2). Also similar to *L-NICD* MCP-1^ΔHep^ mice, CCR2i treatment ameliorated Notch-induced HSC activation and Col1a1 protein levels (Figure 8, D and E), leading to lower liver hydroxyproline content (Figure 8F) and quantitated Sirius red staining (Figure 8G). Overall, these data indicate that Notch-induced MCP-1 secretion contributes to NASH-induced liver fibrosis, which can be blocked by CCR2 antagonists.

## Discussion

Here, we show that Notch-induced MCP-1 is necessary for NASH diet–induced MoMF infiltrate and liver fibrosis and is sufficient to cause both in lean mice. These data suggest a potentially novel hepatocyte/NPC axis. This couples with our previous finding that inhibition of hepatocyte-derived osteopontin in *L-NICD* mice partially rescued fibrosis (20). Although osteopontin and MCP-1 are known fibrogenic factors (32), these studies highlight the central role of hepatocyte/NPC crosstalk to modify the microenvironment in NASH, to coordinate the wound-healing response.

MoMFs typically infiltrate liver tissue during metabolic or toxic damage but are likely dispensable for replenishing the macrophage population in homeostasis, a role attributed to resident Kupffer cells (33–35). MCP-1 is thought to both increase resident macrophage population (9) and promote MoMF infiltration (36). We found no evidence of Kupffer cell deficiency in mice lacking hepatocyte MCP-1, but it is possible that NPC-derived MCP-1 is sufficient to maintain a homeostatic Kupffer cell population in normal liver and that hepatocyte “excess” secretion of MCP-1 preferentially promotes MoMF recruitment. Relatedly, the mechanism of MCP-1–induced liver fibrosis is likely mediated by both increased HSC chemotaxis and proliferation (37) as well as MoMF infiltrate that lead to secondary HSC activation (38, 39). In a potential feed-forward loop, activated HSCs may in turn promote the differentiation of liver macrophages oriented toward profibrotic functions (40). Our data cannot disentangle these 2 potential effects of Notch-induced MCP-1 on liver fibrosis.

A further limitation of our work is that, due to our focus on liver pathology, we have yet to systematically analyze effects of hepatocyte contribution to circulating MCP-1, which may in turn affect other tissues. In humans, serum MCP-1 levels are positively associated with multiple obesity-induced metabolic comorbidities, including type 2 diabetes, as well as serum levels of other inflammatory mediators (41, 42). Given known associations and likely bidirectional contribution of type 2 diabetes to NASH phenotypes (43), we speculate that increased hepatocyte-derived MCP-1 may have systemic impact in other metabolically active tissues (i.e., adipose, muscle), but this requires further study. Another important limitation of our study is that although we found an association between Notch targets and liver *CCL2* expression in patients, all intervention studies were performed in mouse models. Of note, chemokine receptor antagonists have shown sustained and preferential benefit on fibrosis — but not on markers of liver injury and inflammation that constitute the NAFLD Activity Score — in randomized clinical trials in patients with NASH (44). These data support the concept that chemokines may have direct and/or indirect profibrotic effects that may be distinct from widespread changes in liver injury or inflammation. In addition, like many other NASH therapeutics, chemokine receptor antagonists showed wide heterogeneity in treatment response (44). We hypothesize that chemokine antagonists may have preferential effect if applied to patients with advanced liver fibrosis and a Notch-active transcriptional signature on liver biopsy, though this clearly requires formal testing. Despite limitations as discussed, we believe our data provide continued impetus to develop and refine Notch or chemokine inhibitor strategies to treat NASH-associated fibrosis.

## Methods

### Cross-sectional gene expression analysis in patients with suspected NASH.

We analyzed liver gene expression of MCP-1, HES1, HEYL, and JAG1 in 159 individuals who underwent liver biopsy at the Università degli Studi di Milano, Milan, Italy, for suspected NASH due to presence of persistent elevations in liver enzymes or because of severe obesity. Patient characteristics can be found in Supplemental Table 2. The protocol was approved by the Ethical Committee of the Fondazione IRCCS of Milan, and each patient signed a written informed consent form. For statistical analysis, unadjusted univariate analyses were performed. Hepatic MCP-1, HES1, HEYL, and JAG1 mRNA levels were normalized to ACTB expression and natural log–transformed before analyses to ensure a normal distribution.

### Animals.

We crossed homozygous Notch-Venus (24), Rosa^NICD^ (46), or Rosa^DNMAM^ (29) male mice with female C57BL/6J (The Jackson Laboratory, 000664) mice to generate heterozygous transgenic mice for experiments (20). We maintained MCP-1^fl/fl^ (The Jackson Laboratory, 016849) mice on C57BL/6J background. We weaned mice to standard chow (PicoLab rodent diet 20, 5053) for all experiments and started NASH diet (Teklad, TD.160785.PWD) with fructose-containing drinking water (23.1 g of fructose and 18.9 g of glucose dissolved in 1 L of water, filter-sterilized) as indicated in text. We transduced 8- to 10-week-old mice carrying floxed alleles with 1.5 × 10^11^ genome copies of AAV8-*Tbg*-*Cre* (Addgene plasmid AV-8-PV109) or AAV8-*Tbg*-*Gfp* (Addgene plasmid AV-8-PV0146) by tail vein injection. All experiments were performed in male mice, and in a single experimental cohort, unless otherwise stated. Animals were housed in standard cages at 22°C with a 12-hour light/12-hour dark cycle. Upon completion of each study, we weighed and euthanized mice, then collected blood by cardiac puncture. We removed and weighed perigonadal adipose tissues and liver. The Columbia University Institutional Animal Care and Use Committee approved all animal procedures.

### CCR2 antagonist.

We transduced NICD^fl/fl^ mice with AAV8-*Tbg*-*Gfp* or AAV8-*Tbg*-*Cre* to generate control and *L-NICD* mice, then treated with vehicle or CCR2i (ChemoCentryx) (47) by daily oral gavage (30 mg/kg/d) for 2 weeks.

### Plasmids and adenoviruses.

Ad-GFP and Ad-NICD adenoviruses have been previously described (48). We transduced primary hepatocytes at a multiplicity of infection of 10 to achieve 90% to 100% infection efficiency as assessed by GFP-positive hepatocytes. For in vivo MCP-1 overexpression studies, we inserted the mouse MCP-1 coding region into the pLive vector backbone (Mirus, MIR5420), which contains a mouse minimal albumin promoter, using In-Fusion HD Cloning Plus Kit (Clontech, 638909). We dissolved pLive control and pLive–MCP-1 plasmids in TransIT-EE Delivery solution (Mirus, MIR5340), then hydrodynamically injected 20 μg per mouse by tail vein following the manufacturer’s instructions.

### Cell isolation/flow cytometry.

We isolated primary hepatocytes and NPCs as previously described (20, 49). Briefly, we anesthetized mice and digested livers by perfusion of EGTA buffer and collagenase buffer (MilliporeSigma, C5138) through the inferior vena cava, purified hepatocytes with Percoll, and concentrated the remaining NPCs by Nycodenz density centrifugation. We analyzed NPCs by multicolor flow cytometry using an LSRFortessa (BD Biosciences). Briefly, we centrifuged isolated cells at 450*g* for 5 minutes at 4°C, washed in cold staining buffer (PBS, 2% BSA), resuspended 1 × 10^6^ to 10 × 10^6^ NPCs in Zombie Aqua Fixable Viability Dye (BioLegend, 423101) diluted 1:1,000 in PBS, and then incubated for 15–30 minutes at room temperature in the dark. After another wash, we incubated NPCs with TruStain FcX Fc receptor blocker (BioLegend, 101319) for 5 minutes, then with fluorochrome-conjugated antibodies against mouse CD45 (BioLegend, 103157), CD11b (BioLegend, 101239), CD11c (BioLegend, 117329), Ly6C (BioLegend, 128011), Ly6G (BioLegend, 127617), F4/80 (BioLegend, 123130), CD3 (BioLegend, 100236), B220 (BioLegend, 103224), and NK1.1 (BioLegend, 156508) diluted at 1:200 for 20 minutes at 4°C in staining buffer. Gating strategy is shown in Supplemental Figure 1. After staining, we fixed cells with 4% paraformaldehyde for 15 minutes at room temperature, washed, and then resuspended in staining buffer prior to sample acquisition. Total NPCs were further fractionated by FACS, using vitamin A fluorescence of HSCs as previously described (49), or antibody-based cell sorting of lymphoid cells with CD45-APC (BD Biosciences, 559864), myeloid cells with CD11b-FITC (BD Biosciences, 553310), and cholangiocytes with EpCAM-PE (Invitrogen, 12579182). We analyzed data using FCS Express7 (De Novo Software).

### RNA extraction, qPCR, and ChIP.

We extracted RNA with TRIzol (Thermo Fisher Scientific), which we reverse-transcribed to cDNA using the High-Capacity cDNA Reverse Transcription Kit (Applied Biosystems) prior to qPCR with Power SYBR Green (Thermo Fisher Scientific) on a CFX96 real-time PCR detection system (Bio-Rad). Primer sequences are listed in Supplemental Table 3. For ChIP assays, we homogenized 100 mg of liver, fixed and fragmented DNA, and then immunoprecipitated with control IgG or RBPSUH antibodies (Cell Signaling Technology, 5313), followed by PCR with MCP-1 promoter–specific primers as follows: F1-R1: 5′-TCCACAAGCACTTCAGCATGGAGG-3′, 5′-GATAATGGAGGGACTGGGGCCA-3′; F2-R2: 5′-TCCCTTCCAATACTGCCTCAG-3′, 5′-GATAACCCTTCGGGAGAGATAT-3′; and F3-R3: 5′-TCCTGGGGAGTAACAGCATCTAC-3′, 5′-GATAATCAGGCAGCTGAGGTCC-3′.

### Sirius red staining.

We deparaffinized and rehydrated liver paraffin sections, then detected collagen content by Sirius red (Polyscience, 214901) staining per the manufacturer’s protocol. For quantitation, we used 15–20 nonoverlapping slides imaged by a whole-slide scanner (Aperio AT2 scanner) with ImageJ (National Institutes of Health).

### Hydroxyproline measurement.

We measured hydroxyproline content following the manufacturer’s protocol (MilliporeSigma, MAK008). Briefly, liver tissue was homogenized in distilled water and mixed with an equal volume of concentrated hydrochloric acid (~12N HCl), after which homogenates were incubated at 120°C for 3 hours, then oxidized with Chloramine T (MilliporeSigma, MAK008), followed by enzymatic reaction with 4-dimethylaminobenzaldehyde solution. Sample absorbance was measured at 560 nm in duplicate. Hydroxyproline content was expressed as micrograms of hydroxyproline per milligram of liver.

### Luciferase assays.

We generated an MCP-1 promoter-luciferase vector by inserting 3 kb upstream of the coding sequence, which included the predicted Rbpj binding site (mouse: 5′-CTGGGAA-3′ from –2,473 to –2,467), into the pGL4.10 plasmid backbone. We used this plasmid to transfect mouse primary hepatocytes and measured luciferase activity (Promega, E6651) 24 hours after transfection as described (50, 51).

### ELISA.

We assessed secreted MCP-1 from 50 μL conditioned media from Ad-GFP– or Ad-NICD–transduced primary hepatocytes isolated from WT mice using a Mouse MCP-1 ELISA kit (Thermo Fisher Scientific, BMS6005) as per the manufacturer’s protocol.

### Western blots.

We homogenized liver in radioimmunoprecipitation assay buffer with protease and phosphatase inhibitors. We quantified protein and loaded equal amounts for polyacrylamide gel electrophoresis. After blocking, we applied primary antibodies for MCP-1 (Invitrogen, PA1-22488, 1:1,000) and actin (Abcam, ab5694, 1:2,000), then secondary antibodies (MilliporeSigma, GENA934), and quantitated band intensities with either Quantity One (Bio-Rad) or ImageJ.

### Blood analyses.

We measured blood glucose using a glucose meter (Roche, Accu-Chek Aviva Plus) in mice that were fasted for 16 hours.

### Immunofluorescence.

For immunostaining of mouse liver, we incubated frozen slides in HistoVT one (Nacalai Tesque) in a 70°C water bath for 20 minutes. Slides were then incubated at 4°C overnight with primary antibodies against CD45 (BD Biosciences, 550539, 1:100) and CD11b (Abcam, ab133357, 1:100), then secondary antibodies (Thermo Fisher Scientific, A21202 or A21206), prior to mounting with SlowFade Diamond Antifade DAPI Mountant (Thermo Fisher Scientific, S36973). For imaging, we used a ZEISS confocal microscope (Axio Observer Z1 with LSM 710 scanning module).

### Immunohistochemistry.

For immunohistochemistry, sections were dewaxed using xylene for 5 minutes twice and rehydrated with 100% ethanol for 5 minutes, 95% ethanol for 5 minutes, 70% ethanol for 5 minutes, and 50% ethanol 5 minutes, then washed, followed by blocking of endogenous peroxidase activity using 3% hydrogen peroxide. Antigen retrieval process was carried out in a pressure cooker where the slides were immersed in HistoVT one for 20 minutes followed by blocking with 3% BSA, 10% donkey serum. The sections were then incubated with anti-Col1a1 antibody (Cell Signaling Technology, 72026) overnight at 4°C, followed by goat anti-rabbit IgG HRP-conjugated secondary antibody (Cell Signaling Technology, 8114) Diaminobenzidine was used as the chromogenic substrate, and sections were counterstained with Hematoxylin QS (Vector Laboratories, H-3404).

### Statistics.

We expressed results as means ± SEM and calculated differences between 2 groups using a 2-tailed *t* test if the data followed a normal distribution. Analyses involving multiple groups were performed using 1-way ANOVA followed by Tukey’s multiple comparisons test. *P* values of less than 0.05 were considered statistically significant.

### Study approval.

The patient protocol was approved by the Ethical Committee of the Fondazione IRCCS of Milan, and each patient signed a written informed consent form. The Columbia University Institutional Animal Care and Use Committee approved all animal procedures.

## Author contributions

JK, KK, CZ, and UBP developed the study concept and experimental design. JK, JPF, and RJC performed FACS analyses. JK, JY, AB, BM, and DHD conducted in vitro and mouse studies. MM, PD, and LV provided human liver specimens and deidentified pathologic and clinical diagnoses for the patients. JK, JPF, KK, CZ, JY, MM, BM, AB, DHD, AWF, PD, LV, RJC, and UBP analyzed the data. JK and UBP wrote the manuscript, and JK, JPF, KK, CZ, JY, MM, BM, AB, DHD, AWF, PD, LV, RJC, and UBP read and commented on the text and figures.

## Supplementary Material

Supplemental data

## Figures and Tables

**Table 2:**
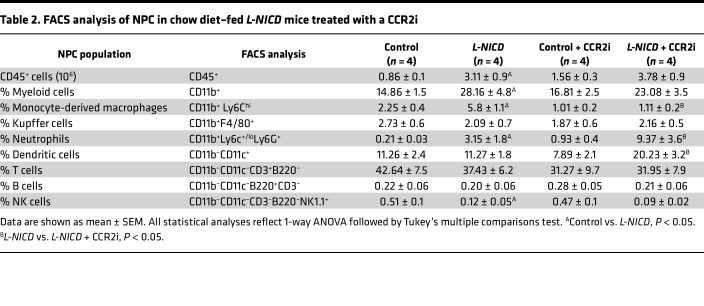
FACS analysis of NPC in chow diet–fed *L-NICD* mice treated with a CCR2i

**Table 1:**
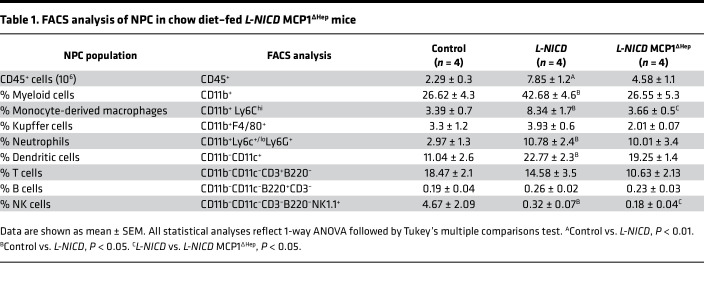
FACS analysis of NPC in chow diet–fed *L-NICD* MCP1^∆Hep^ mice

## Version 1 02/08/2023

Electronic publication
