# Supplementary material for: Notch-mediated hepatocyte MCP-1 secretion causes liver fibrosis
Source: JCI Insight. 2023 Feb 8;8(3):e165369. doi: 10.1172/jci.insight.165369 (PMC9977430; doi:10.1172/jci.insight.165369)
Supplement: Supplemental data [file jciinsight-8-165369-s149.pdf]

**Supplementary Data for:**

# Notch-mediated hepatocyte MCP1 secretion causes liver fibrosis

Jinku Kang[1], Jorge Postigo-Fernandez[1,2], KyeongJin Kim[1,3], Changyu Zhu[1,4], Junjie Yu[1], Marica Meroni[5], Brent Mayfield[1], Alberto Bartolome[1,6], Dianne H Dapito[1], Anthony Ferrante[1], Paola Dongiovanni[5], Luca Valenti[7,8], Remi J. Creusot[1,2] and Utpal B. Pajvani[1]*

**1. Supplementary Figures S1-S6**

**2. Supplementary Tables 1-3**

**Supplementary Figure 1. Characterization of liver immune cell populations in NASH.**

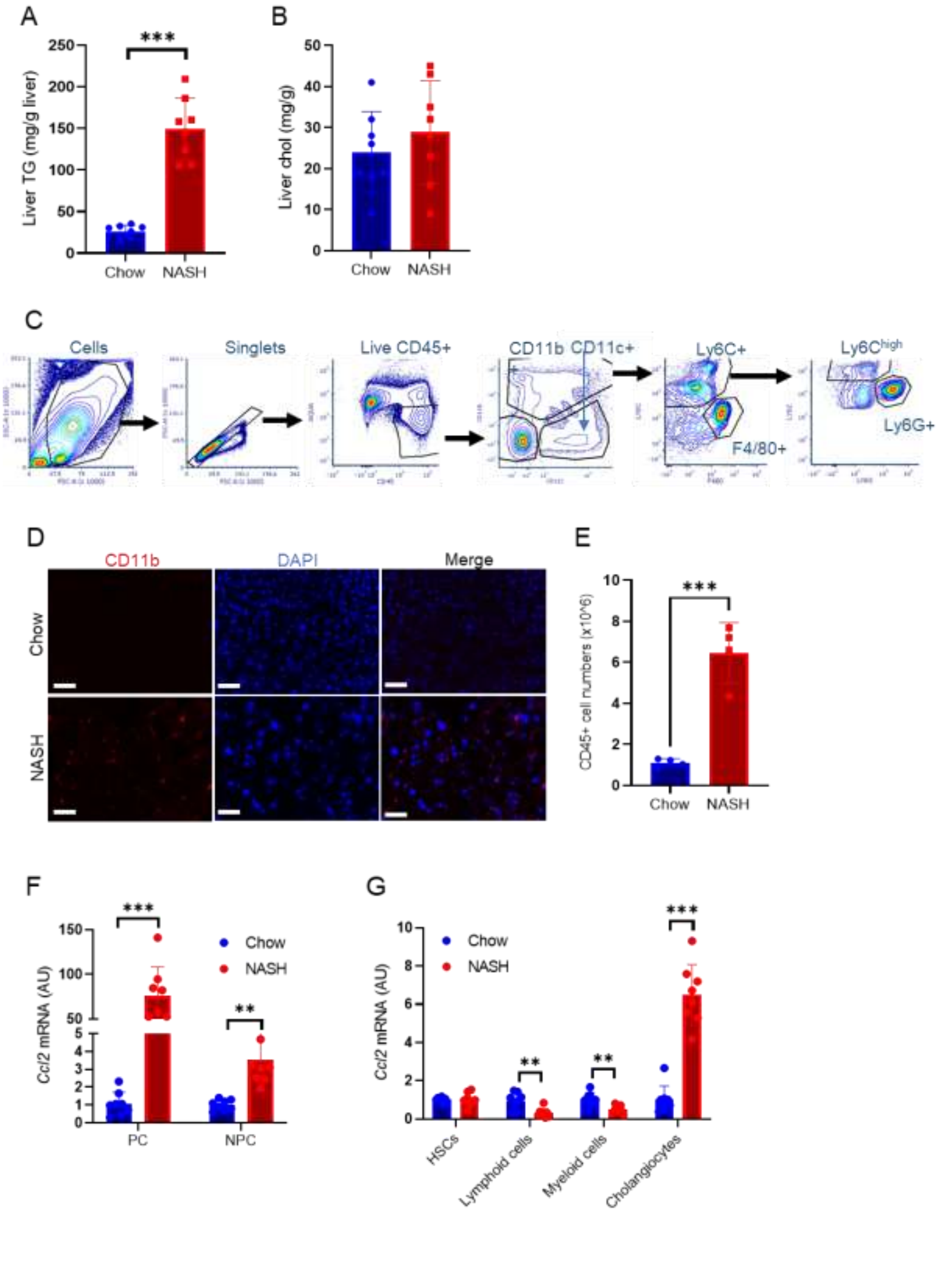

(A) Liver triglyceride and (B) cholesterol levels in chow and NASH male mice liver (N= 8 mice/group). (C) Experimental schematic of workflow to characterize liver immune cell populations. (D) Representative image of CD11b+ cells in livers from chow and NASH diet-fed WT mice. (E) CD45 positive cell number in livers from chow and NASH diet-fed WT mice (N=4 mice/group). (F) *Ccl2* gene expression in fractionated parenchymal cells (PC) and nonparenchymal cells (NPCs) (N=7 mice/group). (G) FACS sorting data of nonparenchymal cells (NPCs) from chow NASH diet-fed WT mice (N= 8 mice/group). Scale bars: 50μm. All data are shown with group means ± s.e.m.; *, $P < 0.05$, **, $P < 0.01$, ***, $P < 0.001$ by two-tailed *t* test.

**Supplementary Figure 2. Further characterization of hepatocyte-specific MCP1 gain-of-function mice.**

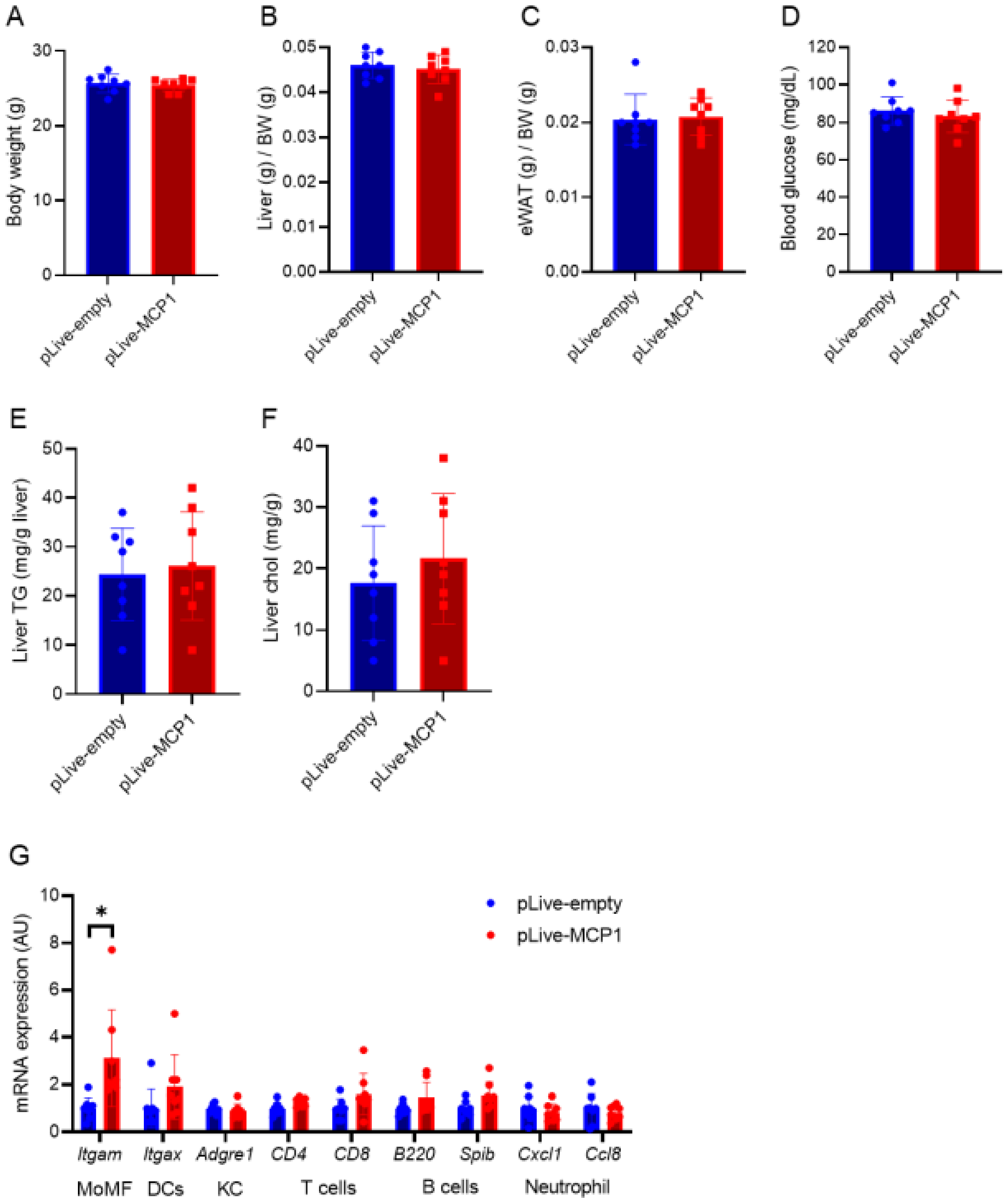

(A) Body weight, (B) liver weight, (C) epidydimal white adipose tissue (eWAT) weight, (D) blood glucose, (E) liver triglyceride, (F) liver cholesterol and (G) gene expression for markers of liver immune cell in chow-fed hepatocyte-specific MCP1 gain-of-function (pLive-MCP1) and control (pLive-empty) WT male mice (N= 8 mice/group). All data are shown with group means ± s.e.m.; *, $P < 0.05$, **, $P < 0.01$, ***, $P < 0.001$ by two-tailed $t$ test.

**Supplementary Figure 3. Further characterization of hepatocyte-specific MCP1 knockout mice.**

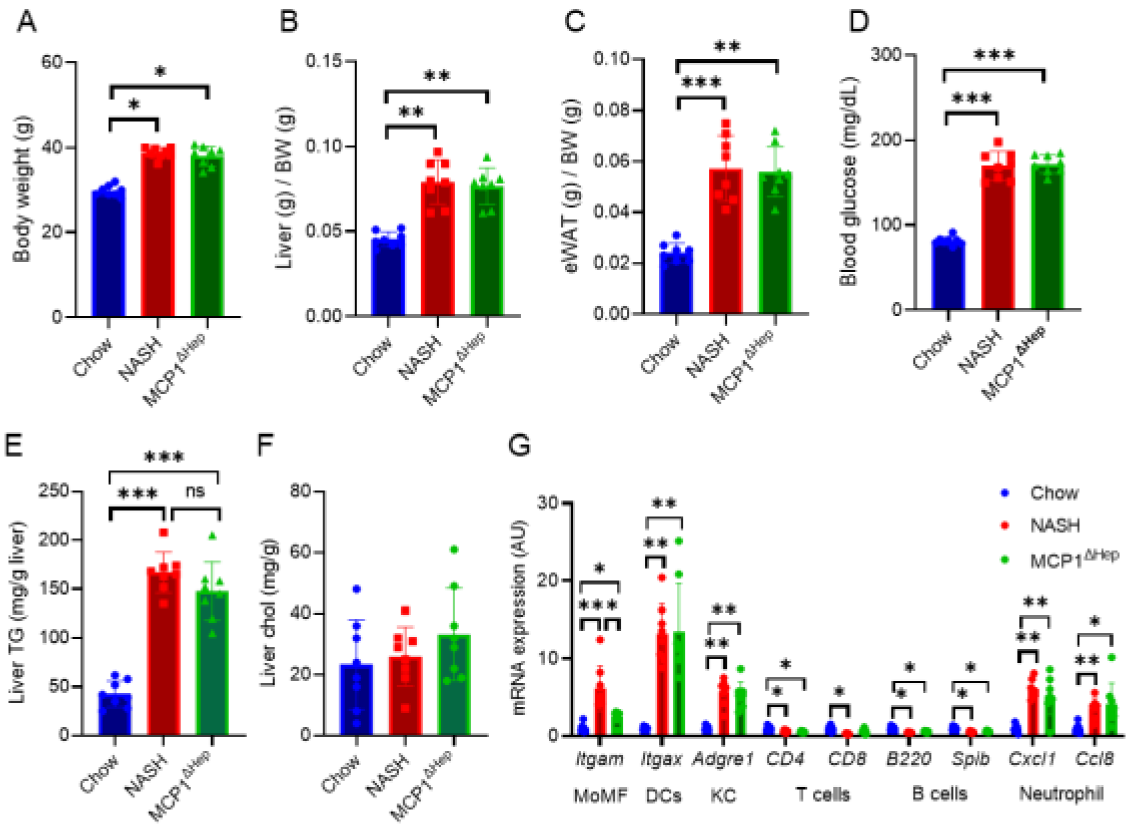


(A) Body weight, (B) liver weight, (C) eWAT weight, (D) blood glucose, (E) liver triglyceride, (F) liver cholesterol in NASH diet-fed control and hepatocyte-specific MCP1 knockout (N=8 mice/group). (G) Gene expression for markers of liver immune cell in NASH diet-fed control and hepatocyte-specific MCP1 knockout (N=8 mice/group). All data are shown with group means ± s.e.m.; *, $P < 0.05$, **, $P < 0.01$, ***, $P < 0.001$ by one-way ANOVA followed by Tukey's multiple comparisons test.

**Supplementary Figure 4. Further characterization of Notch gain- and loss-of-function mice.**

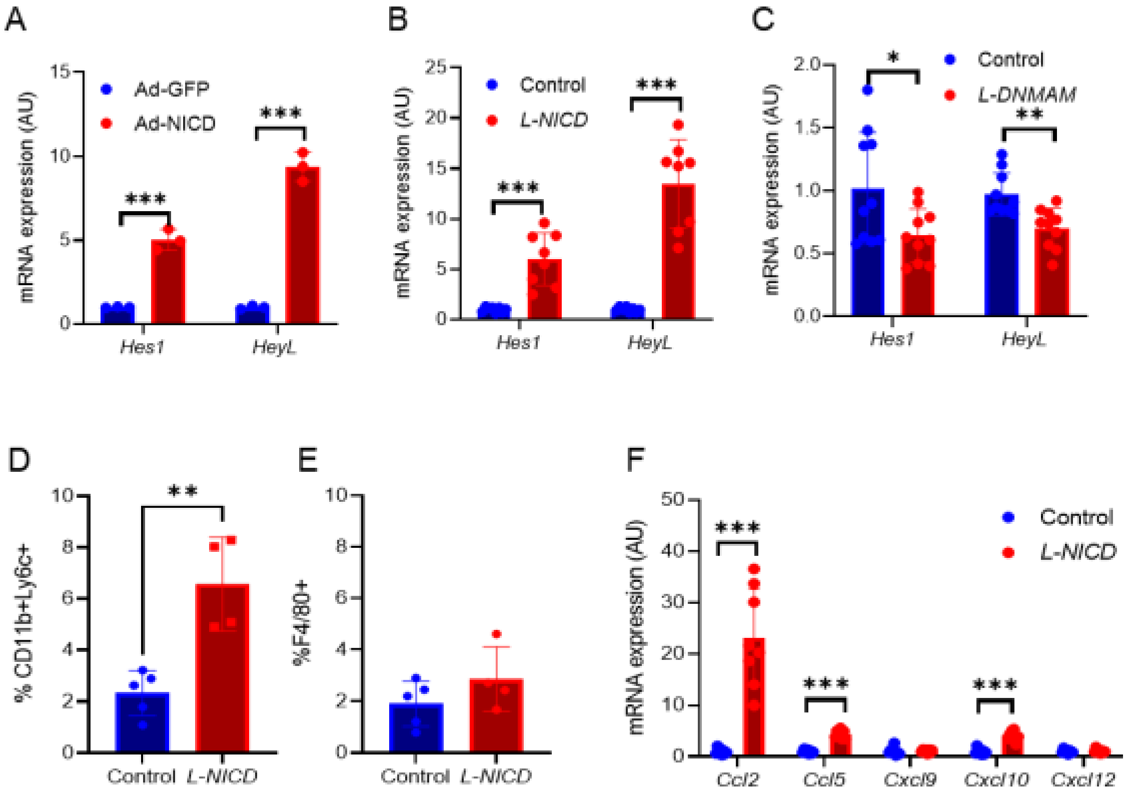


(A) Expression of Notch target genes in Ad-NICD-transduced primary hepatocytes (N=3 biologic replicates/group), and livers from male (B) *L-NICD* (N=8 mice/group) and (C) *L-DNMAM* mice (N=10 mice/group). (D) FACS analysis of CD11b+Ly6C+ and (E) F4/80+ cells from nonparenchymal cells (NPCs) isolated from chow-fed control (Cre-) and *L-NICD* mice (N=4-5 mice/group). (F) Gene expression of hepatic chemokines in control and *L-NICD* male mice (N=8 mice/group). All data are shown with group means ± s.e.m.; **, $P < 0.01$, ***, $P < 0.001$ by two-tailed *t* test.

**Supplementary Figure 5. Further characterization of *L-NICD*:MCP1$^{\Delta Hep}$ mice.**

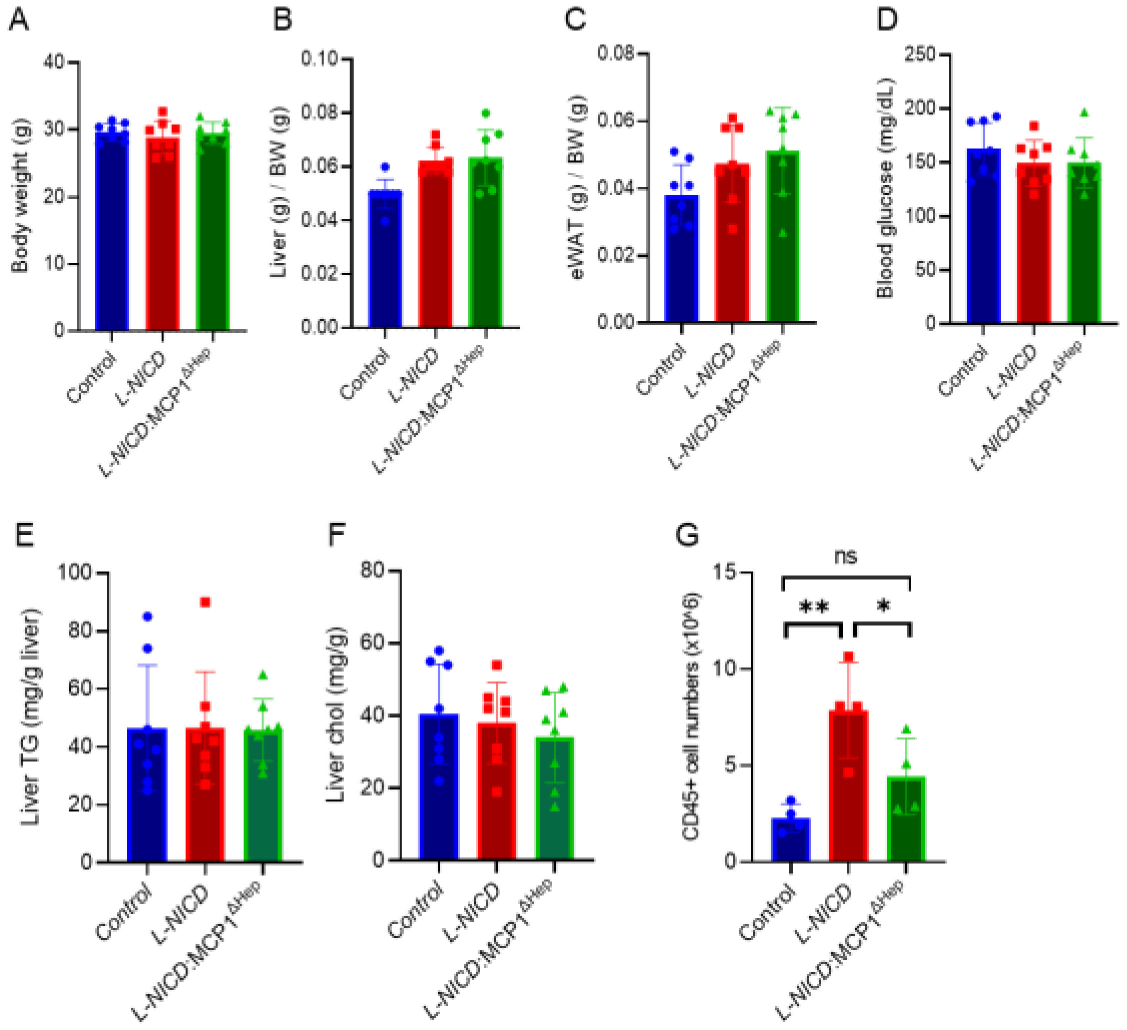


(A) Body weight, (B) liver weight, (C) eWAT weight, (D) blood glucose, (E) liver triglyceride and (F) liver cholesterol in control, *L-NICD* and *L-NICD*:MCP1$^{\Delta Hep}$ male mice (N= 8 mice/group). (G) CD45 positive cell number in control, *L-NICD* and *L-NICD*:MCP1$^{\Delta Hep}$ male mice (N= 4 mice/group). All data are shown with group means ± s.e.m.; *, $P < 0.05$, **, $P < 0.01$, ***, $P < 0.001$ by one-way ANOVA followed by Tukey's multiple comparisons test.

**Supplementary Figure 6. Further characterization of CCR2i-treated *L-NICD* mice.**

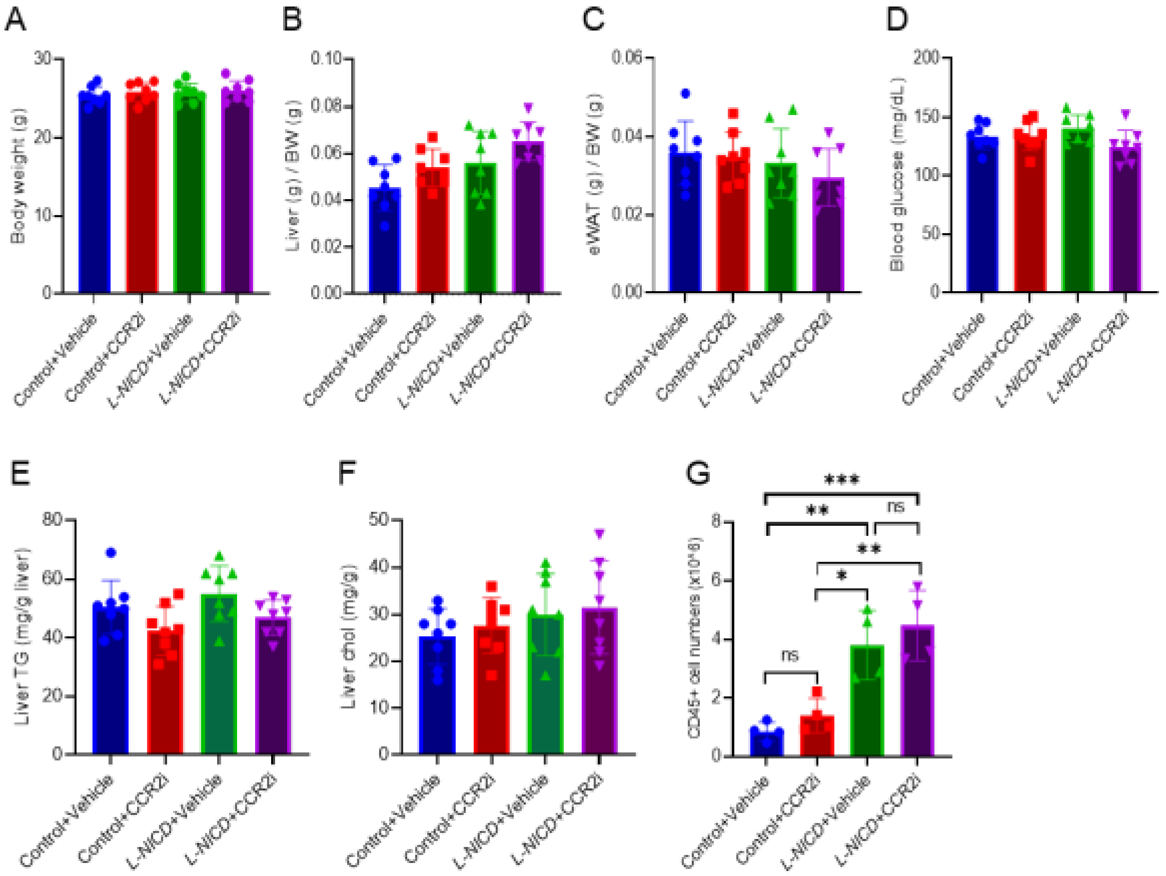


(A) Body weight, (B) liver weight, (C) eWAT weight, (D) blood glucose, (E) liver triglyceride and (F) liver cholesterol in control and *L-NICD* male mice treated with CCR2i or vehicle by daily oral gavage for 2 weeks (N= 8 mice/group). (G) CD45 positive cell number in control and *L-NICD* male mice treated with CCR2i or vehicle by daily oral gavage for 2 weeks (N= 4 mice/group). All data are shown with group means ± s.e.m.; *, $P < 0.05$, **, $P < 0.01$, ***, $P < 0.001$ by one-way ANOVA followed by Tukey's multiple comparisons test.

**Supplementary Table 1. FACS analysis of NPCs in Chow and NASH diet-fed mice.**

| NPC population | FACS analysis | Chow(n=4) | NASH(n=4) |
|---|---|---|---|
| CD45+ cells (x10^6) | CD45+ | 1.09±0.1 | 6.48±0.7** |
| % Myeloid cells | CD11b+ | 22.62±4.3 | 38.68±4.6* |
| % Monocyte-derived Macrophage | CD11b+ Ly6C high | 5.06±2.1 | 11.79±2.1* |
| % Kupffer cells | CD11b+ F4/80+ | 3.26±1.1 | 3.87±1.4 |
| % Neutrophils | CD11b+ Ly6c+/low Ly6G+ | 1.78±0.5 | 3.13±1.2 |
| % Dendritic cells | CD11b- CD11c+ | 13.04±2.6 | 9.77±2.3 |
| % T cells | CD11b-CD11c-CD3+B220- | 25.64±2.1 | 28.51±4.1 |
| % B cells | CD11b-CD11c-B220+CD3- | 0.06±0.008 | 0.07±0.02 |
| % NK cells | CD11b-CD11c-CD3-B220- NK1.1+ | 0.02±0.008 | 0.11±0.2*** |

Data are shown as mean ± SEM. Chow vs NASH:*, $P < 0.05$, **, $P < 0.01$, ***, $P < 0.001$ by two-tailed *t* test.

**Supplementary Table 2. Demographic and clinical features of patients with suspected NASH.**

| **Biopsy cohort (n=159)** | **no-NASH (n=66)** | **NASH (n=93)** | **p value** |
|---|---|---|---|
| Age, years | 44±10 | 46.3±10.4 | 0.16 |
| Sex, female | 48 (73%) | 40 (43%) | 0.0002 |
| BMI, Kg/$m^2$ | 38.7±7.6 | 35.9±9.3 | 0.046 |
| IFG/T2DM | 13 (20%) | 34 (37%) | 0.02 |
| Glucose, mmol/L | 5.3±0.8 | 6.0±2.2 | 0.028 |
| Total cholesterol, mmol/L | 5.3±1.1 | 5.2±1.1 | 0.26 |
| LDL cholesterol, mmol/L | 3.3±0.2 | 2.8±0.1 | 0.02 |
| HDL cholesterol, mmol/L | 1.5±0.9 | 1.4±0.7 | 0.46 |
| Triglycerides, mmol/L | 1.3±0.6 | 1.8±1.2 | 0.01 |
| ALT, IU/L | 21 [16-33] | 46 [26-69] | <0.0001 |
| AST, IU/L | 18 [15-43] | 26 [21-41] | <0.0001 |

Values are means±SD, median [interquartile range], or number (%). BMI: body mass index; IFG: impaired fasting glucose; T2DM: type 2 diabetes mellitus; LDL: low-density lipoprotein; HDL: high-density lipoprotein.

**Supplementary Table 3. Primers used for qPCR.**

| Primers | Organism | Sequence |
|---|---|---|
| *36b4* forward | mouse | 5’-GGAGAAACTGCTGCCTCACA-3’ |
| *36b4* reverse | mouse | 5’-AGCAGCTGGCACCTTATTGG-3’ |
| *Itgam* forward | mouse | 5’-CTCACGTATCCGTGCCTTCTT-3’ |
| *Itgam* reverse | mouse | 5’-GTCCACGCAGTCCGGTAAA-3’ |
| *Col1a1* forward | mouse | 5’-GAACTGGACTGTCCCAACCC-3’ |
| *Col1a1* reverse | mouse | 5’-TTGGGTCCCTCGACTCCTAC-3’ |
| *Hes1* forward | mouse | 5’-ACGACACCGGACAAACCAAA-3’ |
| *Hes1* reverse | mouse | 5’-GAATGCCGGGAGCTATCTTTCT-3’ |
| *Timp1* forward | mouse | 5’-ACTCGGACCTGGTCATAAGGG-3’ |
| *Timp1* reverse | mouse | 5’-CGCTGGTATAAGGTGGTCTCGT-3’ |
| *Tnf* forward | mouse | 5’-CCCACGTCGTAGCAAACCA-3’ |
| *Tnf* reverse | mouse | 5’-TCTTTGAGATCCATGCCGTTGG-3' |
| *Ccl2* forward | mouse | 5'-AGGTCCCTGTCATGCTTCTG -3' |
| *Ccl2* reverse | mouse | 5'-TCTGGACCCATTCCTTCTTG -3' |
| *Ccl5* forward | mouse | 5'-CCCTCACCATCATCCTCACT -3' |
| Ccl8 forward | mouse | 5'-CCAAAGCGGACAGGGATGT-3' |
| Ccl8 reverse | mouse | 5'-GTCCAGGAGCCTTATGGAAGTCT-3' |
| *Ccl5* reverse | mouse | 5'-CCTTCGAGTGACAAACACGA -3' |
| *HeyL* forward | mouse | 5'-AGGTCTTGCAGATGACCGTG-3' |
| *HeyL* reverse | mouse | 5'-CGGGCATCAAAGAACCCTGT-3' |
| *Itgax* forward | mouse | 5'-CGTTCGACACATCCGTGTA-3' |
| *Itgax* reverse | mouse | 5'-TTTGCCTCCTCCATCATTTC-3' |
| *Adgre1* forward | mouse | 5'-CTTTGGCTATGGGCTTCCAGTC-3' |
| *Adgre1* reverse | mouse | 5'-GCAAGGAGGACAGAGTTTATCGTG-3' |
| *Il6* forward | mouse | 5'-AATAACCACCCCTGACCCAAC-3' |
| *Il6* reverse | mouse | 5'-AATCTGAGGTGCCCATGCTAC-3' |
| *Il1b* forward | mouse | 5'-CGCAGCAGCACATCAACAAGAGC-3' |
| *Il1b* reverse | mouse | 5'-TGTCCTCATCCTGGAAGGTCCACG-3' |
| *Il10* forward | mouse | 5'-CCAAGCCTTATCGGAAATGA-3' |
| *Il10* reverse | mouse | 5'-TTTTCACAGGGGAGAAATCG-3' |
| Cxcl1 forward | mouse | 5'-TCTCCGTTACTTGGGGACAC-3' |
| Cxcl1 reverse | mouse | 5'-CCACACTCAAGAATGGTCGC-3' |
| *Cxcl9* forward | mouse | 5'-GAGTGCAAGGAACCCCAGTAGT-3' |
| *Cxcl9* reverse | mouse | 5'-TTGTAGGTGGATAGTCCCTTGGTT-3' |
| *Cxcl10* forward | mouse | 5'-GGATGGCTGTCCTAGCTCTG -3' |
| *Cxcl10* reverse | mouse | 5'-ATAACCCCTTGGGAAGATGG -3' |
| *Cxcl12* forward | mouse | 5'-AGTAGTGGCTCCCCAGGTTT -3' |
| *Cxcl12* reverse | mouse | 5'-GAGACAGTCTTGCGGACACA -3' |
| B220 forward | mouse | 5'-CCCTTCTTCTGCCTCAAAGT-3' |
| B220 reverse | mouse | 5'-CACCTGGATGATATGTGGTCTC-3' |
| Spib forward | mouse | 5'-CTCTGAACCACCATGCTTGC-3' |
| Spib reverse | mouse | 5'-CCCATGTAGAGTCAAGGCCC-3' |
| CD4 forward | mouse | 5'-TCCCACTCACCCTCAAGATA-3' |
| CD4 reverse | mouse | 5'-ATAACCACCAGGTTCACTTC-3' |
| CD8 forward | mouse | 5'-GCCCTTCTGCTGTCCTTGAT-3' |
| CD8 reverse | mouse | 5'-TAGTTGTAGCTTCCTGGCGG-3' |
